# Optimizing prediction of response to antidepressant medications using machine learning and environmental data

**DOI:** 10.1192/j.eurpsy.2021.2000

**Published:** 2021-08-13

**Authors:** A. Spinrad, S. Darki-Morag, D. Taliaz

**Affiliations:** Data Science, Taliaz, Tel Aviv, Israel

**Keywords:** Precision psychiatry, Depression, Treatment optimization, machine learning

## Abstract

**Introduction:**

Major depressive disorder (MDD) is complex and multifactorial, posing a major challenge of tailoring the optimal medication for each patient. Current practice for MDD treatment mainly relies on trial-and-error, with estimated 42%-53% response rates for antidepressant use.

**Objectives:**

We sought to generate an accurate predictor of response to a panel of antidepressants and optimize treatment selection using a data-driven approach analyzing combinations of clinical and demographic factors.

**Methods:**

We analyzed the response patterns of patients to five antidepressant medications in the Sequenced Treatment Alternatives to Relieve Depression (STAR*D) study and the Pharmacogenomic Research Network Antidepressant Medication Pharmacogenomic Study (PGRN-AMPS), and employed state-of-the-art machine learning (ML) tools to generate a predictive algorithm. To validate our results and confirm the algorithm’s external generalizability outside of its training groups, we assessed its capacity to predict individualized antidepressant responses on a separate validation and test sets consisting of 1,021 patients overall from both studies.

**Results:**

The algorithm’s ML prediction models achieved an average accuracy of 0.6416 (64.16%, SD 4.4) across the analyzed medications, and a cumulative accuracy of 0.6012 (60.12%), AUC of 0.601, sensitivity of 0.6034 (60.34%) and specificity of 0.599 (59.9%).

**Conclusions:**

These findings support applying ML to accumulating data derived from large studies to achieve a much-needed improvement in the treatment of depression. By an immediate analysis of large amount of combinatorial data at the point of care, such prediction models may support doctors’ prescription decisions, potentially allowing them to tailor the right antidepressant medication sooner.

**Disclosure:**

Dekel Taliaz is the founder and CEO of Taliaz and reports stock ownership in Taliaz. Amit Spinrad and Sne Darki-Morag serve as data scientists in Taliaz.

